# Whole-genome long-read sequencing to unveil *Enterococcus* antimicrobial resistance in dairy cattle farms exposed a widespread occurrence of *Enterococcus lactis*

**DOI:** 10.1128/spectrum.03672-23

**Published:** 2024-01-17

**Authors:** Medelin Ocejo, Maitane Mugica, Beatriz Oporto, José Luis Lavín, Ana Hurtado

**Affiliations:** 1 Animal Health Department, NEIKER-Basque Institute for Agricultural Research and Development, Basque Research and Technology Alliance (BRTA), Derio, Bizkaia, Spain; 2 Applied Mathematics Department, NEIKER-Basque Institute for Agricultural Research and Development, Basque Research and Technology Alliance (BRTA), Derio, Bizkaia, Spain; US Department of Agriculture, Athens, Georgia, USA

**Keywords:** antimicrobial resistance, dairy cattle, minimum inhibitory concentration, whole-genome sequencing, long-read WGS, *Enterococcus*, *Enterococcus faecalis*, *Enterococcus faecium*, *Enterococcus lactis*

## Abstract

*Enterococcus faecalis* (*Efs*) and *Enterococcus faecium* (*Efm*) are major causes of multiresistant healthcare-associated or nosocomial infections. *Efm* has been traditionally divided into clades A (healthcare associated) and B (community associated) but clade B has been recently reassigned to *Enterococcus lactis* (*Elc*). However, identification techniques do not routinely differentiate *Elc* from *Efm*. As part of a longitudinal study to investigate the antimicrobial resistance of *Enterococcus* in dairy cattle, isolates initially identified as *Efm* were confirmed as *Elc* after Oxford-Nanopore long-fragment whole-genome sequencing and genome comparisons. An *Efm*-specific PCR assay was developed and used to identify isolates recovered from animal feces on five farms, resulting in 44 *Efs*, 23 *Efm*, and 59 *Elc*. Resistance, determined by broth microdilution, was more frequent in *Efs* than in *Efm* and *Elc* but all isolates were susceptible to ampicillin, daptomycin, teicoplanin, tigecycline, and vancomycin. Genome sequencing analysis of 32 isolates identified 23 antimicrobial resistance genes (ARGs, mostly plasmid-located) and 2 single nucleotide polymorphisms associated with resistance to 10 antimicrobial classes, showing high concordance with phenotypic resistance. Notably, linezolid resistance in *Efm* was encoded by the *optrA* gene, located in plasmids downstream of the *fexA* gene. Although most *Elc* lacked virulence factors and genetic determinants of resistance, one isolate carried a plasmid with eight ARGs. This study showed that *Elc* is more prevalent than *Efm* in dairy cattle but carries fewer ARGs and virulence genes. However, *Elc* can carry multi-drug-resistant plasmids like those harbored by *Efm* and could act as a donor of ARGs for other pathogenic enterococcal species.

**IMPORTANCE:**

*Enterococcus* species identification is crucial due to differences in pathogenicity and antibiotic resistance profiles. The failure of traditional methods or whole-genome sequencing-based taxonomic classifiers to distinguish *Enterococcus lactis* (*Elc*) from *Enterococcus faecium* (*Efm*) results in a biased interpretation of *Efm* epidemiology. The *Efm* species-specific real-time PCR assay developed here will help to properly identify *Efm* (only the formerly known clade A) in future studies. Here, we showed that *Elc* is prevalent in dairy cattle, and although this species carries fewer genetic determinants of resistance (GDRs) than *Enterococcus faecalis* (*Efs*) and *Efm*, it can carry multi-drug-resistant (MDR) plasmids and could act as a donor of resistance genes for other pathogenic enterococcal species. Although all isolates (*Efs*, *Efm*, and *Elc*) were susceptible to critically or highly important antibiotics like daptomycin, teicoplanin, tigecycline, and vancomycin, the presence of GDRs in MDR-plasmids is a concern since antimicrobials commonly used in livestock could co-select and confer resistance to critically important antimicrobials not used in food-producing animals.

## INTRODUCTION

Enterococci are opportunistic pathogens present in the intestinal microbiota of animals and humans. Over the last few years, two species, *Enterococcus faecalis* (*Efs*) and *Enterococcus faecium* (*Efm*), have become major causes of multiresistant healthcare-associated and nosocomial infections. Traditionally, the *Efm* population has been classified into two lineages termed clade A, which includes mainly isolates from hospitalized patients (healthcare associated), and clade B, associated with isolates from healthy non-hospitalized individuals (community associated) (1, 2). In 2012, a novel species closely related to *Efm* was described in Italian raw milk cheeses and designated *Enterococcus lactis* (*Elc*) (3). Later on, *Enterococcus xinjiangensis*, isolated from yogurt in China (4) and initially proposed as a novel species, was later confirmed as a heterotypic synonym of *Elc* (5). More recently, Belloso Daza et al. (6) demonstrated by whole-genome sequencing (WGS) analyses that isolates previously classified as *Efm* clade B were in fact members of the new species *Elc* and proposed to reclassify clade B *Efm* as *Elc*. Despite the potential of enterococci to cause disease, some *Enterococcus* strains show probiotic characteristics that make them important candidates for food, human, and animal health applications (7, 8).

The plasticity of the enterococcal genomes allows enterococci to acquire and disseminate antimicrobial genetic determinants of resistance (GDRs). While *Efs* is the most widespread and abundant species of *Enterococcus*, *Efm* is the species that presents higher rates of acquired antimicrobial resistance. Of particular concern are the high-level resistance to ampicillin and the acquired resistance to vancomycin, which are more common in *Efm* than *Efs* (9). Resistant enterococci from animals can infect humans through the food chain or act as a reservoir for antimicrobial resistance genes (ARGs) that can disseminate to other bacteria. Due to their ability to acquire and exchange ARGs, enterococci serve as indicator bacteria for resistance surveillance (10, 11).

With the aim of describing the phenotypic and genotypic resistance profiles of *Efs* and *Efm* isolates of bovine origin, we collected rectal fecal samples from animals of different age groups (calves, heifers, and lactating cows) on five dairy cattle farms in Spain over several samplings. When this study was proposed, clade B members of *Efm* had not been reclassified as *Elc*, and most identification techniques did not differentiate *Efm* clade B from *Elc*. However, midway through this study, the abovementioned reclassification was proposed. We designed a real-time PCR specific for *Efm* (former *Efm*-cladeA) that does not amplify *Elc* (former *Efm*-cladeB) and used it to distinguish both species. Antimicrobial resistance (AMR) of *Efs*, *Efm*, and *Elc* isolates recovered from cattle during the longitudinal study was determined by minimum inhibitory concentration (MIC) against 12 antibiotics, and a selection of genomes was sequenced using Oxford-Nanopore (ONT) technology to identify GDRs and virulence factors (VFs) and perform pangenome analysis. This study has allowed us to identify a wide repertoire of accessory genes that justify the assignment of both former *Efm* clades to different species of the genus *Enterococcus*, design a specific real-time PCR for *Efm*, demonstrate the high prevalence of *Elc* in cattle, and describe the resistance profiles of *Efs*, *Efm*, and *Elc* isolated from cattle.

## RESULTS

### Phylogenomic analyses

A total of 1,070 enterococci colonies resulting from the culture of 107 pools of rectal feces in selective media for *Enterococcus* spp. (10 per plate) were analyzed by the multiplex real-time PCR (RTi-PCR1) amplification assay that simultaneously identifies *Efs* and *Efm* targeting the *mltF* and *ddlA* genes, respectively. Based on RTi-PCR1 amplification results, both species were identified in all farms and age groups, with isolates identified by RTi-PCR1 as *Efm* being more prevalent than *Efs*. Based on these results, 126 isolates (44 *Efs* and 82 *Efm*), that is, one isolate for each *Enterococcus* species per sampling time and age group, were selected for MIC determination.

In all, 32 isolates (17 *Efs* and 15 *Efm*) were then selected for WGS based on the *Enterococcus* species, the phenotypic AMR profile, and the age group. ONT sequencing provided a median of 20,239 reads per sample [interquartile range (IQR) = 15,393–34,315] at a median of 405.4 Mb per sample (IQR = 356.7–654.2 Mb), corresponding to 143× median of coverage value (IQR = 125–226×). The N50 median value was 25,712 bases per sample (IQR = 23,638–29,753). Assembly achieved circularized chromosomes in 29 of the 32 sequenced isolates. In addition, 38 plasmid contigs were identified from 26 isolates, all of which were successfully assembled into complete circular plasmids. Information regarding sequencing output and assembly statistics is provided in Table S1.

**FIG 1:**
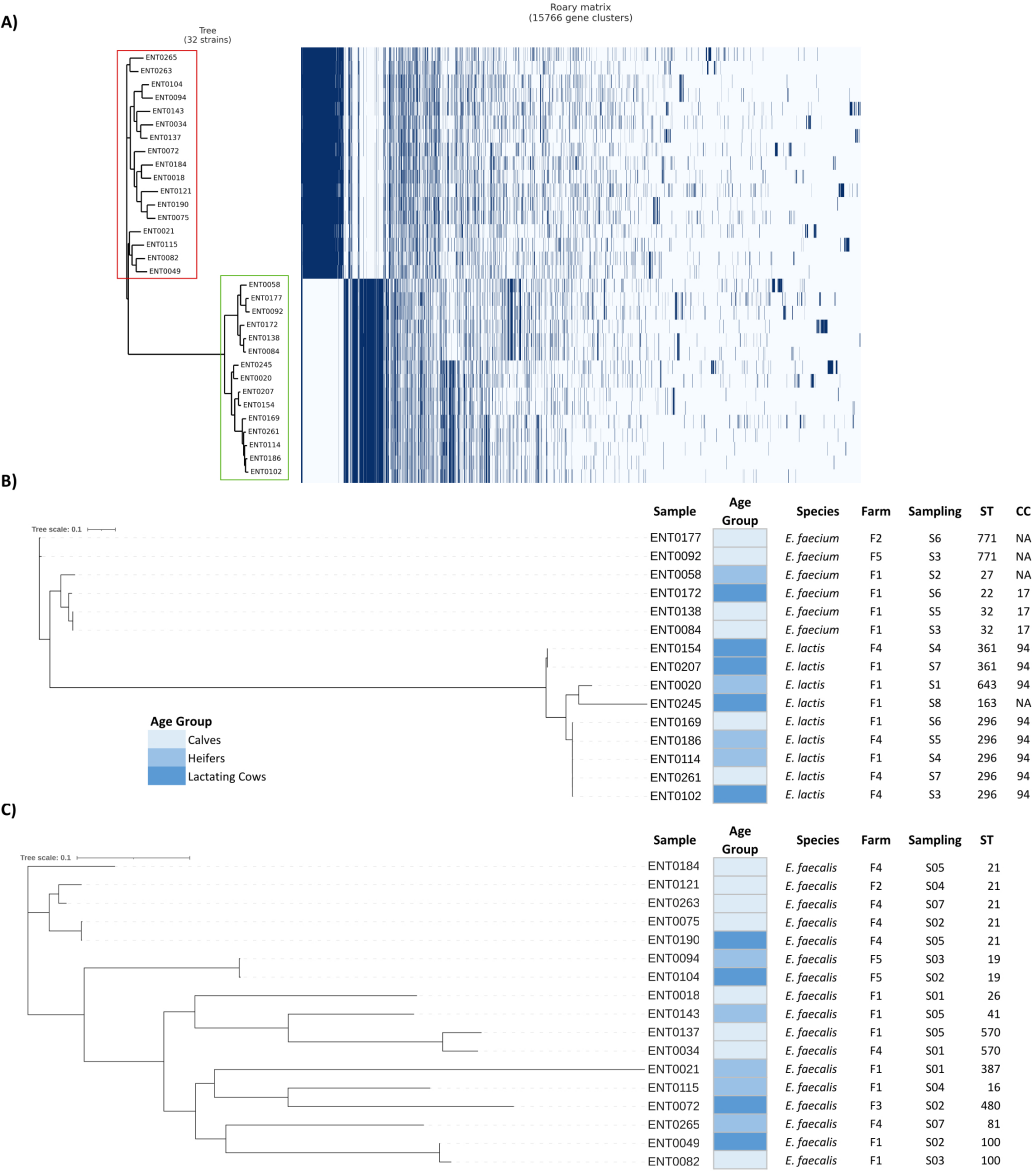
Pangenome analyses of *Enterococcus* genomes. (**A**) Distribution of the 15,766 genes that make up the pangenome across the 32 isolates. The dendrogram represents the hierarchical clustering of the genomes based on the distribution of their accessory genes (presence/absence). The cluster framed in red corresponds to isolates identified as *E. faecalis* and in green as *E. faecium* by RT-PCR1. (**B**) Phylogenetic tree based on the core-genome single nucleotide polymorphisms (SNPs) of *E. faecium* and *E. lactis* (size = 110,229 bases). (**C**) Phylogenetic tree based on the core-genome SNPs of *E. faecalis* (size = 50,251 bases). The phylogenetic trees (B and C) were constructed using Parsnp and RaxML, and corresponding metadata such as age group, farm, sampling time, and MLST results (ST, Sequence Type; CC, Clonal Complex) are indicated for each isolate.

Taxonomic identification results with Kraken2 were in total agreement with RT-PCR1 results. However, the pangenome analysis revealed clear pattern differences between *Efs* and *Efm*, and separated the 15 *Efm* isolates into two subclusters, in both the dendrogram based on the presence or absence of accessory genes (Fig. 1A) and the phylogenetic tree based on core genome alignment (Fig. 1B). Both subclusters shared 13.8% of core genes (1,011/7,314, total size: 110,229 bases). To delve into these findings, genome-scale phylogenetic analysis of the sequenced genomes and the genomes of other *Enterococcus* spp. was performed at the Type (Strain) Genome Server (TYGS) (Fig. S1). This analysis confirmed the identity of the 17 *Efs*. However, the 15 isolates identified by RTi-PCR1 as *Efm* were separated into two clusters: one that included six of our isolates along with other *Efm* isolates belonging to clade A2, and another cluster that included the remaining nine isolates along with clade B *Efm* isolates, *Elc* BT159 (DSM23655; type strain) and *E. xinjiangensis* (JCM30200; heterotypic synonym of *Elc*). The intergenomic comparison between these nine isolates and *Elc* BT159 resulted in a mean DNA:DNA hybridization d_4_ value of 88.8 (range: 83.9–90.3), confirming their classification as *Elc* (by contrast, *E. faecium* NBRC100486 d_4_ = 59.6 [range: 58.2–59.5]). G + C content of both *Elc* BT159 and *E. faecium* NBRC100486 was 38.1%. Based on this phylogenomic analysis, the nine isolates were more closely related to *Elc* than to any other *Enterococcus* species.

### Design and performance of a real-time PCR assay for the specific identification of *E. faecium*

Since RTi-PCR1 did not distinguish *Efm* from *Elc*, a new real-time PCR assay (RTi-PCR2) was designed to amplify the *gluP* gene. RTi-PCR2 produced an amplification signal for *Efm* (former *Efm* clade A) but not for *Elc* (formerly known as *Efm* clade B) or *Efs*, as shown when testing two control strains (kindly provided by Hospital Ramón y Cajal, Madrid) and the field isolates included in this study (6 *Efm* and 9 *Elc*).

**FIG 2:**
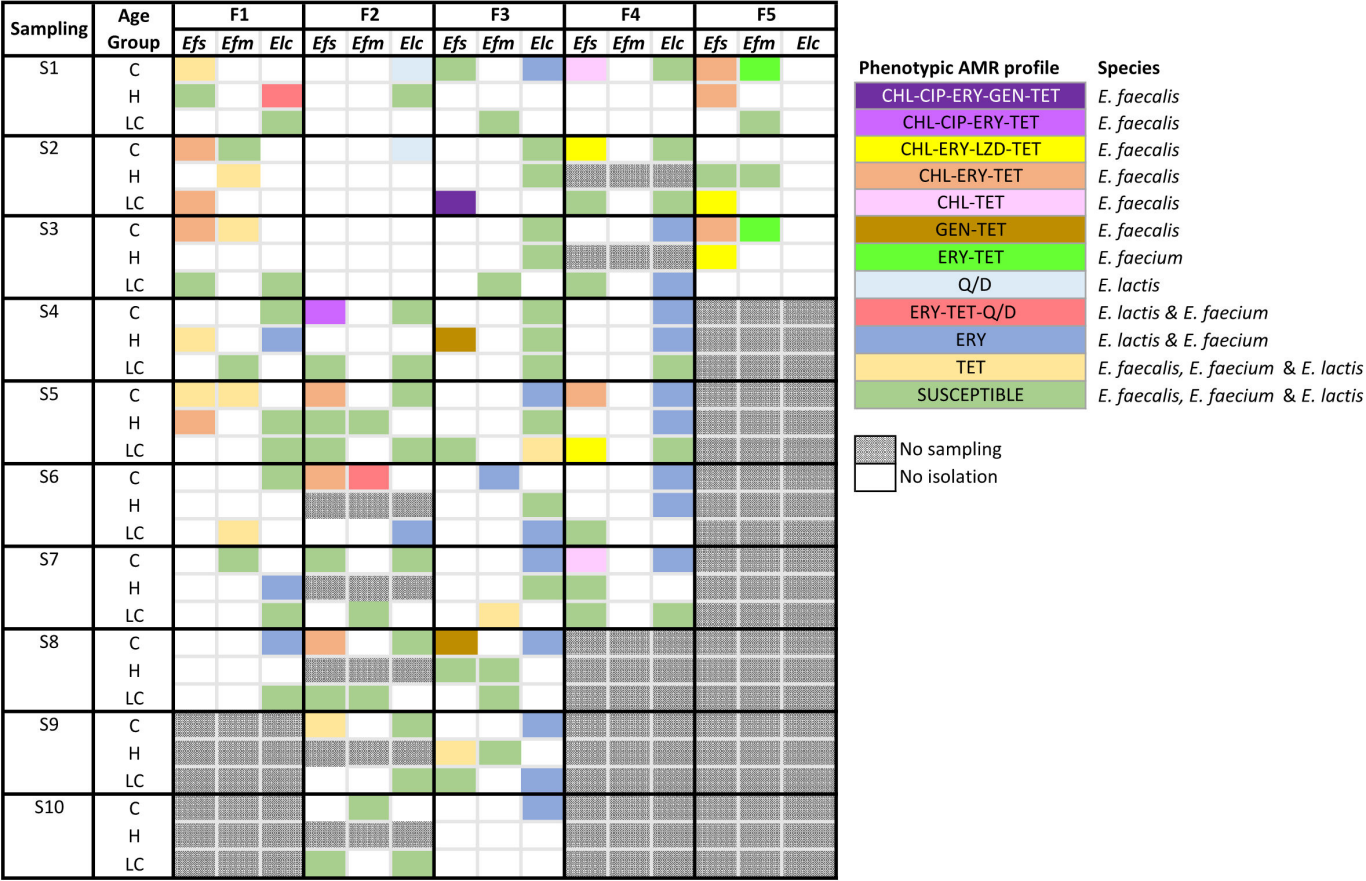
Sampling scheme and distribution of phenotypic AMR profile per farm (F1–F5), sampling (S01–S10), and age group (C: calves; H: heifers; LC: lactating cows). Each AMR pattern is depicted in a different color in the heatmap as described in the legend, which also indicates the enterococci species where each AMR pattern is present. Antimicrobials are abbreviated as follows: CHL, chloramphenicol; CIP, ciprofloxacin; ERY, erythromycin; GEN, gentamicin; LZD, linezolid; Q/D, quinupristin-dalfopristin; TET, tetracycline.

When the 82 isolates identified as *Efm* by RTi-PCR1 were tested using RTi-PCR2, only 23 were confirmed as *Efm*, the remaining 59 being regarded as *Elc* (Fig. 2; Table S2). The isolation frequency of each of the enterococci species did not differ between age groups. Overall occurrence of enterococci (any species) was higher in calves compared to heifers (OR_adj_ = 9.90 (1.80–54.44), *P* = 0.008).

### Antimicrobial resistance as determined by broth microdilution

AMR was phenotypically determined for 126 isolates, that is, 44 (34.9%) *Efs*, 23 (18.3%) *Efm*, and 59 (46.8%) *Elc* (Fig. 2; Table S2). All isolates were susceptible to ampicillin, daptomycin, teicoplanin, tigecycline, and vancomycin. Accordingly, none of the three enterococci species were isolated from the m-Enterococcus plates with vancomycin. Furthermore, a high percentage of isolates (57.6% of *Elc*, 60.9% of *Efm*, and 40.9% of *Efs*) were susceptible to all the antimicrobials tested (Fig. S2A), showing no significant differences in pan-susceptibility among the three species. In general, each species was phenotypically resistant to different antimicrobials. *Efs* showed different levels of resistance to six antimicrobials, that is, 59.1% tetracycline (TET), 43.2% chloramphenicol (CHL), 38.6% erythromycin (ERY), 9.1% linezolid (LZD), 6.8% gentamicin (GEN), and 4.5% ciprofloxacin (CIP). In *Efm* and *Elc,* resistance was restricted to TET, ERY, and quinupristin-dalfopristin (Q/D) (Table S2). Resistance to TET was higher in *Efs* (59.1%) compared to *Elc* (3.4%; OR_adj_ = 41.17 (8.89–90.64), *P* < 0.001) and in *Efm* (34.8%) compared to *Elc* (OR_adj_ = 15.20 (2.92–79.19), *P* = 0.001). All resistant *Efs* isolates showed resistance to TET either alone or in combination with 1–4 other antimicrobials. In *Efs*, ERY-resistant isolates (38.6%) always showed high MIC_ERY_ values (>128 mg/L) and a multi-drug resistance (MDR) profile (always including ERY-CHL-TET). Conversely, the majority of ERY-resistant *Elc* isolates (20/22) had MIC_ERY_ values just above the ECOFF (8 mg/L) and were mostly susceptible to all other antimicrobials. ERY-resistance in *Efm* accounted for 17.4% (4/23) of the isolates, three of them with a high MIC_ERY_ value (>128 mg/L). A few isolates showed MIC_Q/D_ > 4 mg/L, that is, 1 *Efm* (4.3%) and 3 *Elc* (5.1%).

Overall resistance rates were higher in calves (67.9%) followed by heifers (43.3%) and lactating cows (25.6%) (Fig. S2B), differences between calves and lactating cows being statistically significant (OR _adj_ = 7.24 (2.66–19.70), *P* < 0.001). In *Efs*, statistically significant differences were observed between age groups for certain antimicrobials. Isolates from calves showed higher resistance to CHL than those from heifers (OR_adj_ = 15.49 (1.33–180.93), *P* = 0.029) and lactating cows (OR _adj_ = 6.69 (1.18–37.81), *P* = 0.032). Similarly, *Efs* were more resistant to TET when isolated from calves than from heifers (OR_adj_ = 23.40 (1.69–324.19), *P* = 0.019) or lactating cows (OR_adj_ = 39.13 (3.62–422.78), *P* = 0.003).

MDR, defined as resistance to at least three antimicrobial classes, was mainly found in *Efs* (38.6% of isolates) recovered from all the farms (Fig. S2B), with 55.6% of the *Efs* isolates from calves, 27.3% from heifers and 26.7% from lactating cows being MDR. The only phenotypic MDR patterns present in all age groups were CHL-ERY-LZD-TET and CHL-ERY-TET. In addition, the profile CHL-ERY-TET was present in four of the five farms (Fig. 2). Farm F5 showed the highest percentage of MDR *Efs* isolates (83.3%) and farm F3 the lowest (12.5%) despite harboring the only isolate resistant to five antimicrobial classes.

### Genomic composition of the isolates

**FIG 3:**
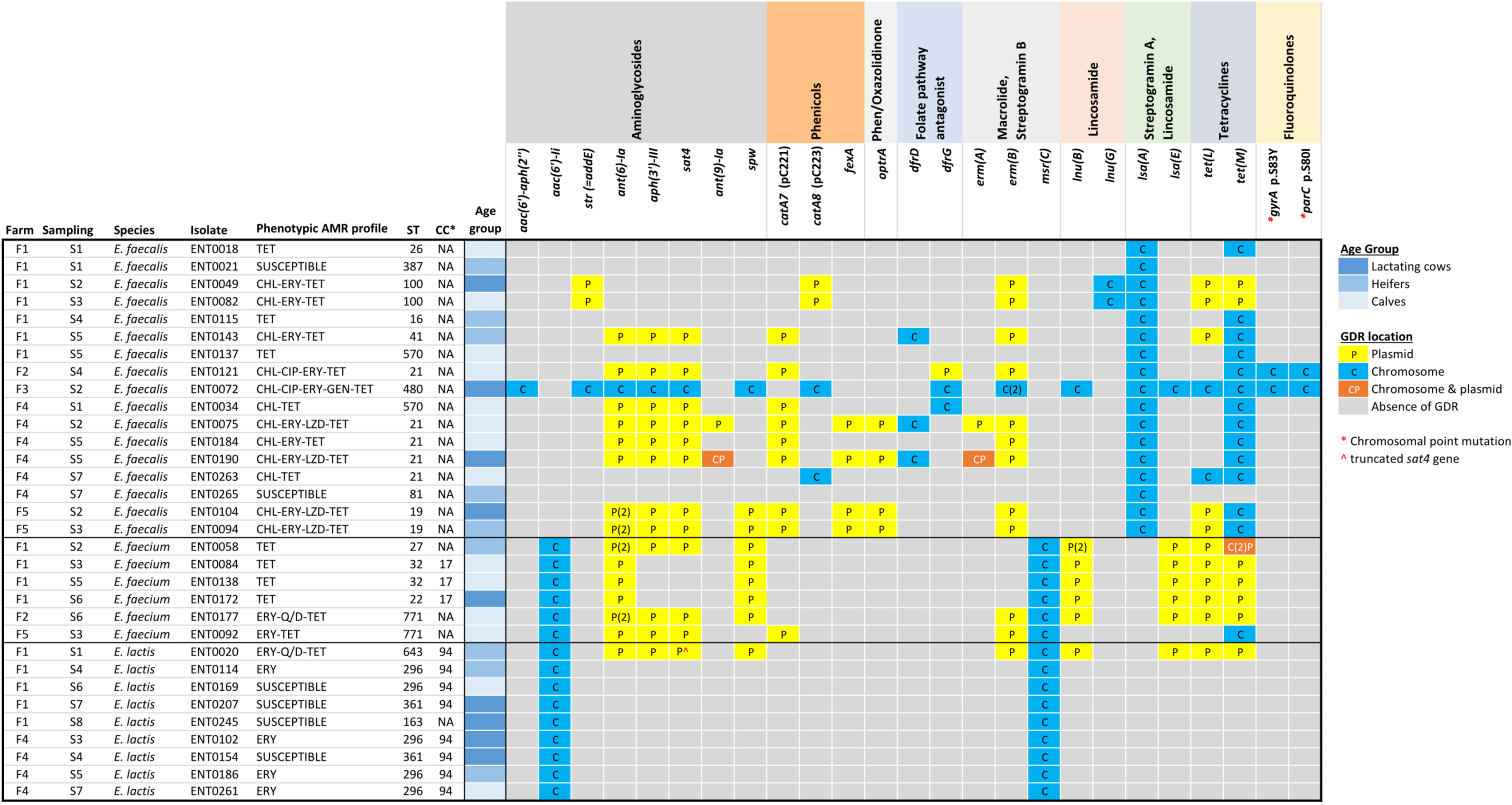
Heatmap of the distribution of the AMR genes detected by WGS. The presence and location of the GDRs are indicated as per the legend.

The 32 genomes were assigned to 18 MLST types (STs), that is, 17 *Efs* belonged to 10 STs, six *Efm* to four STs, and nine *Elc* to four STs (Fig. 3). The most prevalent MLST type in *Efs* was ST21 (*n* = 5), two STs were detected twice (ST32 and ST771) in *Efm*, and ST296 predominated in *Elc* (*n* = 5). *Efm* and *Elc* did not share STs, and when assigned to a clonal complex, *Efm* isolates belonged to CC17 and *Elc* isolates to CC94 (Fig. 3). The phylogenetic tree based on the core genome single nucleotide polymorphisms (SNPs) was consistent with the MLST results (Fig. 1B and C).

Plasmids were identified in 13 *Efs* isolates (18 plasmids), 6 *Efm* (12 plasmids), and 7 *Elc* (8 plasmids) with variable sizes, ranging from 9.8 to 211.8 Kbp (Table S1). More than half of the detected plasmids (21/38) contained ARGs (2–10 each), corresponding to 10 *Efs* isolates (14 plasmids), six *Efm* (6 plasmids), and one *Elc* (1 plasmid). In fact, the majority of acquired ARGs were located in plasmids (76.1%), the main exception being *tet*(M) in *Efs* (Fig. 3).

A total of 79 virulence genes (71 chromosomally encoded, seven only present in plasmids, and one in both) were identified. The percentage of identity shared was above 90% for most genes from enterococci (*n* = 55) and ranged between 64.1% and 80.1% for genes from other Gram (+) bacteria (*n* = 24) (Table S3). VFs were more abundant in *Efs*, with isolate ENT0115 carrying the largest number of VFs, including the aggregation substance (AS) and the cytolysin operon, which were not found in the other isolates. Although *Efm* and *Elc* shared most of their VFs, seven of the nine *Elc* carried the nidogen-binding LPXTG surface adhesin gene *sgrA*, which was not identified neither in *Efm* nor *Efs*.

### Detection of genes and chromosomal point mutations associated with antimicrobial resistance

In all, 25 different GDRs (23 ARGs and two chromosomal point mutations) that encode resistance to antimicrobials belonging to 10 different classes were identified, resulting in 18 different genotypic resistance profiles (Fig. 3). *Efs* genomes carried the largest number of GDRs (*n* = 23), 10 of them also present in *Efm* and *Elc*. Genes intrinsically present in the chromosome of all isolates were *lsa*(A) (resistance to lincosamides and streptogramin A) in *Efs*, and *aac(6′)-Ii* (resistance to tobramycin, kanamycin, and amikacin) and *msrC* (low-level resistance to ERY and quinupristin) in both *Efm* and *Elc*. Only one *Elc* isolate (ENT0020) carried acquired ARGs (nine ARGs in a plasmid), and all of them were also detected in *Efm*.

The two SNP mutations were only detected in two *Efs* and conferred resistance to fluoroquinolones: one in the *gyrA* gene (S83Y) and another in the *parC* gene (S80I). In addition, PointFinder identified several mutations in the Penicillin-Binding Protein 5 (PBP5) sequences of six *Efm* and one *Elc* (ENT0020) corresponding to intermediate PBP5-S/R hybrid types (Table S4), but none presented the consensus PBP5-R allele.

ARGs encoding aminoglycoside resistance were the most abundant and diverse (Fig. 3). The only isolate resistant to GEN, an MDR *Efs* isolated from lactating cows in F3 (ENT0072), carried the *aac(6′)-aph(2*″) gene in the chromosome. Two genes encoding streptomycin (STR) resistance were detected, that is, *str* in *Efs* (*n* = 3 isolates) and *ant(6)-Ia* in the three species (*n* = 16). The *aph(3′)-III* gene was also found in the three species (*n* = 13) as part of the *ant(6)-Ia*―*sat4*―*aph(3′)-III* aminoglycoside-streptothricin resistance gene cluster. The spectinomycin-resistant gene *ant(9)-Ia* was present in two *Efs* isolates (in plasmids in both and with a second copy in the chromosome in one) and it was always present in the same plasmid or chromosomal region as the *erm*(A) gene (ERY). All isolates with MIC_ERY_ ≥128 mg/L, that is, two *Efm*, one *Elc*, and 10 *Efs,* harbored the *erm*(B) gene, generally located in a plasmid. However, four other ERY-resistant *Elc* (MIC_ERY_ = 8 mg/L, three isolates and MIC_ERY_ = 16 mg/L, one isolate) did not carry the *erm*(A) or *erm*(B) genes.

Determinants of CHL resistance were mostly restricted to *Efs* and included *catA8* (*cat*_pC223_) (*n* = 4) and *catA7* (*cat*_pC221_) (*n* = 9); *catA8* (*cat*_pC223_) was present in plasmids in two (duplicate) isolates and the chromosome in another two unrelated isolates, whereas *catA7* (*cat*_pC221_) was always located in plasmids along with several other ARGs. The only *Efm* isolate (ENT0092) carrying a CHL resistance gene, *catA7* (*cat*_pC221_), did not show phenotypic resistance to CHL (MIC_CHL_ = 32 mg/L, one dilution step below the ECOFF). Four *Efs* isolates were resistant to both CHL and LZD and besides *catA7* (*cat*_pC221_), they all carried the *fexA* 688 nt upstream of the *optrA* gene in other plasmids.

The *lsa*(E) gene, associated with resistance to lincosamides and streptogramin A antibiotics, was detected in the chromosome of one *Efs* and in plasmids of five *Efm* and one *Elc*. Genes that encode the nucleotidyl transferase and confer resistance to lincosamides were also detected, that is, *lnu*(B) in *Efm* (*n* = 5) and *Elc* (*n* = 1), and *lnu*(G) in *Efs* (*n* = 2). Trimethoprim resistance-associated genes were only detected in *Efs*, *dfrD* (*n* = 3) and *dfrG* (*n* = 3). Finally, all TET-resistant isolates harbored the *tet*(M) gene (*n* = 22), 13 of them along with the *tet*(L) gene. The *tet*(M) gene was generally chromosomally encoded in *Efs* and plasmid-encoded in *Efm* and *Elc*, whereas *tet*(L) was mostly located in plasmids in all species (Fig. 3).

### Within-farm transmission dynamics of resistant enterococci

In some instances, duplicate isolates (same ST, GDRs, and VFs) were found during several samplings infecting animals of different age groups within a farm (Fig. 3). This was the case of two ST19 *Efs* isolated from calves and heifers during two consecutive samplings in F5 and two *Efs* isolates of ST100 recovered from calves and lactating cows in F1. In *Efm*, isolates of ST32 (recovered at samplings S03 and S05 from calves in F1) shared their phenotypic and genotypic AMR profiles and carried identical VFs. On the other hand, ST21, the most abundant MLST type in *Efs*, was identified in F2 (one isolate) and F4 (four isolates) and all presented a different content of ARGs. Similarly, the two *Efs* isolates belonging to ST570, also from F1 and F4, were different in their gene content and antimicrobial susceptibility. In *Efm*, the two ST771 isolates (F2 and F5) carried a different repertoire of plasmid-encoded ARGs. In *Elc*, isolates with the same genomic profile were isolated from different farms. Thus, ST361, isolated from lactating cows in F1 and F4, was identical in all the examined features, and ST296, found in all age groups and two farms (F4, *n* = 3 and F1, *n* = 2), shared their GDR content (*aac(6’)-Ii* and *msrC* genes) even though four isolates showed MICs for ERY above the ECOFF (MIC_ERY_ = 8–16 mg/L but lacking an ERY-associated GDR).

## DISCUSSION

This study was aimed at describing the within-farm dynamics and AMR profiles of *Efm* and *Efs* isolated from animals of different age groups (calves, heifers, and lactating cows) in dairy cattle farms. However, WGS and pangenome analysis of a selection of isolates showed that several isolates initially identified as *Efm* were in fact *Elc*, an *Enterococcus* species first isolated from Italian raw milk cheese (3). Like several recent reports (12, 13), our results confirm the proposed re-classification of *Efm* clade B as *Elc* (6). Consequently, the former division of *Efm* between commensal- and healthcare-associated lineages would now be the result of two different species. Upon analysis of the genome sequences, we verified the presence of the *ddlA* gene in the genomes of both *Efm* and *Elc*, confirming the amplification of both species with RTi-PCR1. Considering that most identification techniques available did not differentiate *Efm* clade A from *Elc*, we designed a new real-time PCR assay for the specific identification of *Efm* (only the formerly known clade A). Protocols for the specific PCR identification of *Elc* have already been described (14, 15) but here we developed a TaqMan real-time PCR assay that uses a specific hydrolysis probe for the detection of *Efm*. The primers and probe designed here can be used in a duplex format with the *mltF*-targeting primers and probe (16) to simultaneously detect *Efs* and *Efm* (data not shown). When used to re-analyze all the isolates initially identified as *Efm* with RTi-PCR1, the study herein showed a widespread occurrence of *Elc* in dairy cattle.

Resistance to antibiotics considered critically or highly important to human medicine has been described to be low in enterococci recovered from cattle (10, 17). However, resistant enterococci can easily exchange GDRs with other enterococci and Gram-positive bacterial species and contribute to the spread of resistance (18). In this study, resistance was more frequent in *Efs* than in *Efm* and *Elc* but all isolates were susceptible to ampicillin, vancomycin, teicoplanin, tigecycline, and daptomycin. Enterococci produce chromosomally encoded low-affinity penicillin-binding proteins (PBPs) that bind weakly to β-lactam antibiotics resulting in reduced susceptibility to penicillin. The most important mechanism for high-level ampicillin resistance in *Efm* is specific amino acid changes in the PBP5 sequence (19). Here, even though six *Efm* and one *Elc* showed intermediate PBP5-S/R hybrid types, none contained the PBP5 profile associated with ampicillin resistance. This agrees with previous studies that showed that strains associated with animals (primarily of subclade A2) displaying ampicillin MICs in the range 0.5–128 mg/L, mainly harbored hybrid-like PBP5 (PBP5-S/R) alleles (20). In fact, beside the allelic type, the ampicillin resistance phenotype is related to the PBP5 expression levels which are likely associated with differences in the upstream region of *pbp5* (21). Acquired (enhanced) resistance to ampicillin is rare in *Efs* but common among clinical *Efm* isolates. The prevalence of AMP resistance in *Efm* of animal origin varies depending on the region and host, and in cattle is lower than in poultry or swine (10, 22). Similarly, vancomycin resistance in enterococci isolated from food-producing animals is more common in *Efm* than *Efs*, and isolation of vancomycin-resistant *Efm* is more frequent in poultry and pigs compared to cattle (22). Here, vancomycin-resistant isolates were not recovered even when selective isolation in vancomycin-containing media was performed.

LZD, the first oxazolidinone antibiotic, is a critically important antimicrobial used to treat vancomycin-resistant enterococci. Resistance is associated with ribosomal mutations or the acquisition of the *cfr*, *poxtA* or *optrA* genes (23). Here, *Efs* of two different MLST types (ST19 in F5 and ST21 in F4) that shared the same MDR phenotypic profile (CHL-ERY-LZD-TET) showed LZD resistance. They carried the oxazolidinone-phenicol transferable resistance gene *optrA* that codes for an ABC transporter and confers a decreased susceptibility to oxazolidinones and phenicols in plasmids of different sizes but always co-localized with the *fexA* gene. Co-carriage of additional ARGs in the same plasmid is of particular relevance for the dissemination of *optrA*-carrying plasmids even when LZD is not used (not approved for use in food-producing animals) (23). The use of florfenicol in farm animals may select for florfenicol-resistant bacteria that also carry the *optrA* gene (24). Although LZD resistance is still rare in humans, *Efs* harboring transferable *optrA* and *poxtA* genes is spreading in Spain (25). Similarly, the increasing levels of resistance in enterococci recovered from food-producing animals and food of animal origin reported in different countries, mainly associated with *optrA* gene carriage (22, 23, 26), is a concern. Resistance to CHL was widespread in *Efs*, and all resistant isolates sequenced carried variants of the *cat* gene that code for chloramphenicol acetyltransferase enzymes. As previously reported (22), the *catA7* (*cat*_pC221_) gene variant was more prevalent, whereas the *catA8* (*cat*_pC223_) gene was more sporadically detected. Interestingly, the four LZD-resistant isolates that carried the *optrA* and *fexA* genes also carried *catA7* (*cat*_pC221_) but in a different plasmid.

Resistance to ERY (MIC_ERY_ >4 mg/L) was moderate in *Efm* and similarly high in *Efs* and *Elc*. However, MIC values among ERY-resistant isolates were higher in *Efs* and *Efm* compared to *Elc*, and only isolates with MIC_ERY_ >128 mg/L carried the *erm*(B) gene. Conversely, most ERY-resistant *Elc* isolates showed MIC_ERY_ values just above the ECOFF and those sequenced lacked any ERY-associated GDR. The *erm*(B) gene, that confers resistance to macrolide-lincosamide-streptogramin B antibiotics, is the most common genetic determinant of ERY resistance and is widespread in human and animal isolates (22). Q/D, a mixture of streptogramin A (dalfopristin) and B (quinupristin), is effective against *Efm* but not against *Efs* (27) that intrinsically carry the *lsa*(A) gene (28). In *Efm*, resistance against streptogramin A-type antibiotics can be mediated by the PLS_A_ resistance gene *lsa*(E) (29) or *via* enzymatic inactivation by the acetyltransferases VatD and VatE (30). Resistance to streptogramins B is either encoded by the *erm*(B) gene or the *vgbA* gene, which encodes a staphylococcal-type lactonase (31). Here, *vat* and *vgb* genes were not detected but the presence of the *erm*(B) and *lsa*(E) genes (1 *Efm* and 1 *Elc*) was associated with higher MIC values for Q/D (MIC_Q/D_ = 8 mg/L). The presence in isolates from cattle of plasmids carrying both *Isa*(E) and *erm*(B) genes is a concern since Q/D is a valuable alternative to vancomycin for the treatment of multi-drug-resistant *Efm* infections.

Enterococci have intrinsic low-level resistance to aminoglycosides due to their low cell wall permeability or inactivation by naturally occurring enterococcal enzymes (32). For example, all *Efm* and *Elc* isolates tested in this study carried a chromosomally encoded 6′-acetyltransferase enzyme (AAC(6′)-Ii) capable of modifying aminoglycosides like tobramycin, kanamycin, and amikacin (33, 34). Therefore, only two aminoglycosides (GEN and STR) are not readily affected by intrinsic enzymes produced by enterococci and are reliably used in clinical practice (for synergism with β-lactams) (2). In the present study, a high level of resistance to GEN (MIC_GEN_ >1,024) was observed in three *Efs* isolates from the same farm (F3, recovered from calves, heifers, and lactating cows). Of them, the only isolate subjected to WGS carried the bifunctional gene *aac(6′)-aph(2″*), the most important aminoglycoside-modifying enzyme encoding gene since it confers resistance to most of the clinically available aminoglycosides (e.g., GEN, tobramycin, amikacin, kanamycin) but not STR (32, 35). On the other hand, the *ant(6)-Ia* gene (mostly within the aminoglycoside-streptothricin cluster *ant(6)-Ia*―*sat4*―*aph(3′)-III*) was widespread among the sequenced isolates indicating a high rate of STR-resistance. Other acquired aminoglycoside resistance genes detected in our panel of isolates included *ant(9)-Ia*, *aph(3′)-III*, *str*, *sat4*, and *spw*.

TET resistance was low in *Elc* but high in *Efm* and very high in *Efs*. The higher prevalence of TET resistance in *Efs* was associated with the widespread presence of *tet*(M), mainly located in the chromosome. Conversely, in *Efm* and *Elc*, *tet*(M) was mostly encoded in plasmids along with *tet*(L). All MDR isolates were resistant to TET, an antimicrobial widely used in livestock that might co-select enterococci resistant to other antimicrobial agents. Genes associated with trimethoprim resistance (*dfrD* and *dfrG*) were occasionally present in *Efs*; however, since enterococci can utilize exogenous sources of folate inhibitors trimethoprim is ineffective *in vivo* (36). Although fluoroquinolones are commonly used in dairy cattle, here, resistance to CIP was low and only found in two *Efs* isolates, in both cases associated with mutations in the *gyrA* and *parC* genes, the most common mechanism of resistance (37).

Horizontal gene transfer and the remarkable genome plasticity of enterococci play a key role in their evolution and survival, and the acquisition and dissemination of GDRs. The most frequently reported mechanism for foreign DNA acquisition in enterococci is conjugation *via* conjugative plasmids and conjugative transposons (or integrative conjugative elements) (2). Here, most isolates carried the majority of acquired resistant genes in plasmids, with up to 10 ARGs in a single plasmid. *Efs* isolate ENT0072 was the exception since it carried on the chromosome 13 different ARGs and SNP mutations in the *gyrA* and *parC* genes.

Isolates with identical MLST type, ARG content, and plasmids were occasionally obtained from consecutive samplings within the same farm suggesting that the same strain persisted in the farm over time infecting animals of different age groups. This was the case with the LZD-resistant isolates, which were detected in different samplings and age groups in two farms. Exceptionally, within the same farm isolates of the same ST carried different ARG, mainly in plasmids but also in the chromosome (e.g., *Efs* ST21 in F4 isolated over 10 months in calves and lactating cows), indicating the frequent occurrence of gene exchange. By contrast, isolates from different farms were in general distinct and even when genetically related (same MLST type), they generally carried different ARGs in different plasmids. Also noteworthy was the presence of multiple copies of certain ARGs in several isolates, either in the chromosome, in a plasmid, or both. Notably, one *Efm* isolate carried two copies of *tet*(M) in the chromosome and another in a plasmid. Conjugative elements such as the Tn916/1545 and Tn5385 transposon families, commonly associated with *tet*(M) (38), could have mediated a recombination event. This redundancy of determinants of resistance has been described in settings under high selective pressure.

Although *Efs* and *Efm* are opportunistic pathogens, *Elc* has probiotic characteristics. The pathogenic potential of *Enterococcus* spp. is based on their adhesion capacity, their ability to evade the immune system, and their potential to form biofilms. VFs were more widespread in *Efs* and similarly distributed among *Efm* and *Elc* isolates. The isolate with the largest number of VFs (*n* = 46) was an *Efs*. Interestingly, it was the only one that carried the cytolysin operon (Cyl), that encodes a secreted two-peptide lytic protein that damages host cells and promotes infection (39), and the aggregation substance AS, a pheromone-induced surface protein that facilitates plasmid conjugation and potentiates the pathogenic effect of Cyl (40). Regarding *Elc*, they all had a MIC_AMP_ ≤2 mg/L and lacked the virulence genes IS16, *hyl*, and *esp*, thus meeting the criteria set by the European Food Safety Authority (EFSA) to consider a strain safe (41). However, one *Elc* (ENT0020) carried eight ARGs in a plasmid, which poses a risk of transmitting resistance to other bacteria. Besides, seven *Elc* isolates carried the *sgrA* gene that codes for a nidogen-binding LPXTG surface adhesin. SgrA binds to extracellular matrix proteins and is involved in biofilm formation (42). The two *Elc* that did not carry *sgrA* (ENT0020 and ENT0245) were the most divergent isolates within the *Elc* cluster in the pangenome analysis.

In conclusion, the identification of enterococci at the species level has clinical relevance due to differences in pathogenicity and antibiotic resistance profiles. Traditional methods did not distinguish *Elc* from *Efm*. Even commonly used taxonomic classifiers like Kraken2 failed to correctly assign the corresponding species due to the misidentification of the reference strains included in the database. Therefore, the interpretation of data on *Efm* epidemiology and characterization from previous studies biased by *Elc* misidentification should be reconsidered. The *Efm* species-specific real-time PCR assay developed here will help to properly identify *Efm* (only the formerly known clade A) in future studies. Here, we showed that *Elc* is prevalent in cattle. Whole-genome characterization showed that GDRs and VFs were more widespread in *Efs* than in *Efm*, and GDRs were more abundant in both species compared to *Elc*. Although *Elc* most probably poses a lower human health hazard than *Efm* or *Efs*, some isolates can carry MDR plasmids similar to those harbored by *Efm* and could act as a donor of ARGs for other pathogenic enterococcal species. Although all isolates were susceptible to critically or highly important antibiotics like daptomycin, teicoplanin, tigecycline, and vancomycin, as well as ampicillin, resistance to LZD (*Efs*) and Q/D (*Efm* and *Elc*) was detected in a few isolates. Co-carriage of several ARGs in MDR-plasmids is a concern since antimicrobials commonly used in livestock could co-select and confer resistance to critically important antimicrobials not used in food-producing animals. Further studies are in progress to deeply characterize the mobilome and ARG synteny of the chromosomes sequenced in this study.

## MATERIALS AND METHODS

### Sampling design

Five dairy cattle farms in the Basque Country were selected to study the within-farm diversity and dynamics of *Enterococcus* spp. and their resistance profiles. The Basque Country is a 7,234 km^2^ region located in northern Spain, where 18,126 dairy cattle are currently managed under an intensive production system according to the 2020 census (https://en.eustat.eus). The commercial farms enrolled in the study (designated F1–F5) were representative of the style of farming in the region. Samplings, initially planned on a monthly basis for 1 year, extended between 2019 and 2020 due to the COVID-19 pandemic and consisted of 7–10 samplings in four of the farms (F1–F4) and three in another (F5). At each sampling time, rectal fecal samples were collected from five apparently healthy animals from each of three age groups (1- to 5-month-old calves, 5- to 22-month-old heifers, and lactating cows) and analyzed in a single 25 g pool per age group (5 g per animal). At five sampling visits in F2 and two in F4, heifers were not available for sampling. Thus, a total of 535 rectal fecal samples were collected and analyzed in 107 pools. Samplings were carried out by veterinary practitioners in strict accordance with Spanish ethical guidelines and animal welfare regulations (RD 53/2013) as part of their routine veterinary practice, and therefore, ethical review and approval of the Ethics Committee for Animal Experimentation was not required. Informed oral consent was obtained from the farmers at the time of sampling.

### *Enterococcus* selective isolation

Feces (a total of 25 g) were diluted 1:10 in Brain Heart Infusion (BHI, Difco Laboratories, Detroit, MI, USA) broth supplemented with sterile NaCl 6.5% and incubated at 37 ± 1°C for 18 to 24 h. Subsequently, 20 µL of the incubated broth was subcultured onto m-Enterococcus Agar (Difco Laboratories) for the selective isolation of *Enterococcus* spp. Simultaneously, for the selective isolation of vancomycin-resistant enterococci (VRE), 10 µL of the initial broth was subcultured on BHI broth supplemented with 2 mg/L vancomycin (Sigma-Aldrich, Saint Louis, MO, USA). After incubation (37 ± 1°C for 18 to 24 h), 20 µL was subcultured onto m-Enterococcus plates with vancomycin (6 mg/mL) and incubated at 37 ± 1°C for 18 to 24 h. Ten colonies per plate were selected based on colony morphology and subcultured onto Blood Agar (Columbia agar + 5% sheep blood, bioMérieux, Marcy-l’Etoile, France) for further identification of *Efs* and *Efm* by the simultaneous real-time PCR amplification of the *mltF* gene of *Efs* and the *ddlA* gene present in *Efm* (RTi-PCR1), as described elsewhere (16).

### Design of a real-time PCR assay for the specific identification of *E. faecium*

A real-time PCR (RTi-PCR2) amplification assay was developed for the amplification of the *gluP* (rhomboid protease) gene for the specific detection of *Efm*. The *gluP* gene was selected based on the pangenome analyses performed by Belloso Daza et al. (14); this gene is present in *Efm* and *Elc* but only shares a 90%–92% homology. Aligned sequences of the *gluP* gene of 16 *Efm* and 34 *Elc* were compared for primer and probe design, and an *in silico* specificity analysis was then performed for each primer and probe by submitting their nucleotide sequences against the GenBank databases using BlastN. Thus, a pair of primers (*Efm*-gluP-F: 5′- ACATAACCCAGCGATCCAG-3′; *Efm*-gluP-R: 5′- CCAATTAGCCCACCGACAT-3′) and a probe (FAM─5′-CCCAAACAT/ZEN/CTACAGAGGTATCCAGAAGAC-3′─Iowa Black FQ; Integrated DNA Technologies, Coralville, Iowa, USA) were designed to specifically amplify a 120 bp fragment of the *gluP* gene of *Efm* but not *Elc*. A QuantStudio 5 real-time PCR system (ThermoFisher Scientific, Waltham, MA, USA) was used to perform the RTi-PCR using 96-well (0.2 mL) consumables. Reactions were performed in a volume of 15 µL and included 1× Premix Ex Taq (TaKaRa Bio, Mountain View, CA, USA), 0.5× ROX Reference Dye II (TaKaRa Bio), 0.3 µM of each primer, and 0.2 µM of probe. Cycling consisted of a denaturation step of 30 s at 95°C, followed by 35 amplification cycles of 95°C for 15 s and 60°C for 1 min. Negative (sterile water) and positive controls were included in each real-time PCR amplification assay. Threshold values (Ct) below 32 cycles were considered positive and those greater than 32 cycles were considered uncertain.

### Antimicrobial susceptibility test determination by broth microdilution

MIC values were determined for 12 antimicrobial agents belonging to 10 classes using Sensititre EUVENC antimicrobial susceptibility test (AST) Plates (ThermoFisher Scientific), following the recommendations in Commission Implementing Decision 2020/1729/EU regarding antimicrobials and dilution ranges. The results were interpreted using epidemiological cut-off (ECOFF) values defined by EUCAST (European Committee on Antimicrobial Susceptibility Testing; http://www.eucast.org) to discriminate microorganisms with and without acquired resistance mechanisms (non-wild-type resistant and wild-type susceptible, respectively). *Efs* are intrinsically resistant to pleuromutilins, lincosamides, and streptogramins A (the so-called PLS_A_ phenotype) due to the expression of the *lsa*(A) gene (28) and, therefore, MIC results for Q/D were not interpreted. For *Efm* and *Elc*, a MIC of >1 mg/L was considered as a reference following EFSA recommendations (43) but only those with MIC_Q/D_>4 mg/L were interpreted as resistant.

### Whole-genome sequencing, genome assembly, and bioinformatics analysis

Genomic DNA was extracted with NZY Microbial gDNA Isolation kit (NZYTech, Lisbon, Portugal) and it was quantified and quality-assessed using a NanoDrop 1000 spectrophotometer (ThermoFisher Scientific) and a Qubit 2.0 Fluorometer (ThermoFisher Scientific). WGS was carried out using Oxford Nanopore Technology (ONT, Oxford, UK) for long-read sequencing. Library preparation was performed following the genomic DNA ligation protocol (SQK-LSK109), and sequencing was carried out on a MinION MK1C device (ONT) using FLO-MIN106D (R9.4.1) and FLO-MIN111 (R10.3) flow cells (ONT). The output files generated by ONT sequencing were base-called in high accuracy mode and quality-filtered using Guppy v.5.0.13 (Qscore >8). Sequencing adapters were removed using Porechop v.0.2.4 with default parameters (44) and Filtlong v.0.2.0 (https://github.com/rrwick/Filtlong) was employed to filter sequences based on their size and quality. Reads with a size smaller than 1,000 base pairs (bp) were discarded, and a subset comprising the top 90% of reads, as determined by their quality score (Q), was selected. In samples where the sequence data were larger than 1,000 Mbp, lower quality reads were progressively removed until obtaining 1,000 Mbp of sequences.

Subsequently, sequences were *de novo* assembled using Unicycler v.0.4.8 (44). Chromosome- and plasmid-derived contigs were predicted using PlasFlow v.1.1 (45). Species identification was performed using Kraken2 v.2.0.9 (46). Multi-locus sequence types (MLST) were inferred using Krocus v.1.0.3 (47) unassembled long-reads. Genomes were then screened for acquired AMR genes using ABRicate v.1.0.1 (T. Seemann, https://github.com/tseemann/abricate) against the ResFinder database (updated 18/01/2022) (48) for acquired AMR genes and hits were filtered at 90% coverage and identity. Chromosomal point mutations associated with AMR were identified by screening unassembled reads against the Pointfinder database (updated 28/03/2022) (49) using the Resfinder tool v.4.1.0 (50). VFs were identified using ABRIcate against the full version of the Virulence Factors Data Base (VFDB_setB_nt.fas.gz, updated 11/05/2023). Virulence genes were filtered at 85% coverage and 60% identity, and the pattern of presence/absence of these genes was used to generate a dendrogram. The hierarchical clustering analysis for the dendrogram was performed with the unweighted pair-group method with arithmetic mean (UPGMA) based on the Jaccard distance matrix, using the function hclust (v.3.6.1) of the R statistical package v.3.6.3. The presence of IS16 was investigated by screening the genomes against a custom database containing the target sequence downloaded from ISFinder (51) using ABRIcate.

Isolates from the same source (farm) that exhibited the same MLST type, VFs, and GDR patterns were considered as duplicate isolates; isolates from different farms sharing these features were considered the same strain. When distinguishable by any of these features they were referred to as different strains.

### Pangenome analysis

Chromosomic pangenome analysis was conducted to investigate the genetic diversity and evolutionary relationships among the *Enterococcus* isolates in the European Galaxy server (https://usegalaxy.eu/). Genomes annotation was performed using Prokka v.1.14.6 (52), and the pangenome was calculated using Roary v.3.13.0 (53), employing a minimum blastp identity threshold of 95% for gene family definition. Genes from the Roary output were categorized as core genes (present in 99%–100% of the isolates), soft-core genes (95%–99%), shell genes (15%–95%), and cloud genes (0%–15%). In addition, a matrix was generated to illustrate the gene presence and absence patterns across the different species, along with the dendrogram that illustrates the relationship among isolates based on the accessory genes.

For the classification and identification at the species level of our isolates, genome-based taxonomy was performed at TYGS (Type Genome Server), a server that infers genome-scale phylogenies and state-of-the-art estimates for species and subspecies boundaries from user-defined and automatically determined closest type genome sequences through pairwise genome comparisons (54). To infer the phylogenetic relationships among the *Enterococcus* isolates, parsnp v1.7.4 (55) along with the implemented RaxML v.8.2.12 (56) was used to construct phylogenetic trees based on core genome SNPs, with default parameters and specifying -r! parameter to randomly select the reference from the set of genomes analyzed. The resulting trees were visualized and edited with iTOL v.6.8 (https://itol.embl.de).

### Statistical analysis

Multivariate logistic regressions were performed to statistically examine the differences among various variables in relation to age groups and/or *Enterococcus* species. These variables encompassed (i) the overall distribution of enterococci, (ii) the distribution of each specific *Enterococcus* species, (iii) the occurrence of phenotypical AMR to each of the studied antimicrobial agents, (iv) pan-susceptibility, and (v) MDR, defined as resistance against three or more antimicrobial classes. Odds ratios (OR), along with their corresponding 95% confidence intervals (95% CI), were employed as a measure of association between positivity and the explanatory variables (age groups and *Enterococcus* species), adjusted for the variable of farms. Differences were considered statistically significant if *P* < 0.05.

## Data Availability

Raw sequencing data of the 32 strains analyzed in this study are available at the NCBI Sequence Read Archive (SRA) database under accession numbers as detailed in Table S5, associated with the BioProject PRJNA1024516.

## SUPPLEMENTAL MATERIAL

Supplemental material spectrum.03672-23-s0001.xlsx Figures S1 and S2 and Tables S1-S5.

ASM does not own the copyrights to Supplemental Material that may be linked to, or accessed through, an article. The authors have granted ASM a non-exclusive, world-wide license to publish the Supplemental Material files. Please contact the corresponding author directly for reuse.
